# Nanoparticle Behavior in the Body: Biochemical, Pharmacological and Cellular Aspects

**DOI:** 10.3390/nano16191195

**Published:** 2026-09-22

**Authors:** Rodion G. Sakhabeev, Arina V. Ilina, Inga A. Gorbacheva, Elizaveta N. Sezganova, Anna S. Rogova, Aleksandra V. Sagaidak, Dmitry O. Vinokhodov, Maxim Shevtsov

**Affiliations:** 1 St. Petersburg State Institute of Technology, Moskovsky Ave. 26, 190013 St. Petersburg, Russia; arina-ilina-23@yandex.ru (A.V.I.); militisss@yandex.ru (I.A.G.); sezganova2003@gmail.com (E.N.S.); aleksandrasagaidak@yandex.ru (A.V.S.); vinokhodov@list.ru (D.O.V.); 2 Saint Petersburg State University, Universitetskaya Emb. 7/9, 199034 St. Petersburg, Russia; 3 Peter the Great St. Petersburg Polytechnic University, Polytechnicheskaya St. 29, 195251 St. Petersburg, Russia; anna.aroo@mail.ru; 4 Laboratory of Biomedical Nanotechnologies, Institute of Cytology of the Russian Academy of Sciences (RAS), Tikhoretsky Ave., 4, 194064 St. Petersburg, Russia; 5 Department of Radiation Oncology, Technische Universität München (TUM), Klinikum rechts der Isar, Ismaninger Str. 22, 81675 Munich, Germany

**Keywords:** nanoparticles, metabolism, pharmacokinetics, protein corona, mononuclear phagocyte system, intracellular transport, immunomodulation

## Abstract

The behavior of nanoparticles (NPs) in the body is a complex, multilevel process governed by a cascade of physicochemical and biological interactions. At the molecular level, these processes include protein adsorption and formation of the biomolecular corona, ion release, and surface redox reactions. At the cellular level, they encompass endocytosis, intracellular trafficking, lysosomal processing and clearance, and redistribution within cellular compartments. At the systemic level, NPs undergo tissue and organ biodistribution, biological barrier crossing, biotransformation, biodegradation, elimination, and immunomodulation. Understanding these processes is essential for evaluating the safety, efficacy, and therapeutic potential of nanomedicines, as the unique physicochemical properties of NPs can induce biological responses that differ substantially from those of their bulk counterparts. This review provides an integrated framework for understanding the absorption, distribution, biotransformation, and elimination of NPs in biological systems. Particular emphasis is placed on the molecular and cellular mechanisms governing nanoparticle–biological interactions, including the effects of NPs on cellular energy metabolism through interactions with the mitochondrial electron transport chain and their immunomodulatory effects mediated by nuclear factor κB (NF-κB) signaling and inflammasome activation. By integrating molecular, cellular, and systemic perspectives, this review highlights the key determinants of nanoparticle fate in the body and their implications for the design, safety, and clinical translation of nanomedicines.

## 1. Introduction

Over the past two decades, nanoparticles have become an important component of diagnostic and therapeutic strategies, including targeted drug delivery, imaging, and tissue engineering [1,2]. Their small size (1–100 nm), high specific surface area, and reactivity [3,4,5] enable modulation of the pharmacokinetics and pharmacodynamics of therapeutic agents and help overcome the limitations of conventional dosage forms [6,7]. However, these same properties make the behavior of NPs in biological environments difficult to predict. Assessment of the toxic properties of nanomedicines remains a critical aspect of their evaluation [8]. Unlike small-molecule compounds, NP dosing cannot be based on mass alone: the biological response is determined by particle number, surface area, size, shape, and surface charge [6,8]. Physiologically based pharmacokinetic (PBPK) modeling can integrate these parameters; however, conventional PBPK models developed for small molecules and based on passive diffusion are not applicable to NPs because their distribution is governed by phagocytosis, endocytosis, and transcytosis, necessitating the development of specialized nano-PBPK models [9].

Nanomedicine development must account for a set of specific factors that determine NP behavior in the body. First, protein corona formation is of central importance. This dynamic layer of adsorbed proteins alters the biological identity, biodistribution, half-life, and performance of NPs [10]. Corona composition depends both on the physicochemical properties of the particles themselves—such as size, hydrophobicity [11], and charge [12]—and on host-specific characteristics, including sex [13]. Second, recognition of NPs by the mononuclear phagocyte system (MPS), particularly by hepatic and splenic macrophages, constitutes a critical barrier and results in rapid clearance from the bloodstream [14]. Third, in addition to size and charge, relevant physicochemical parameters include shape, crystallinity, aggregation stability, and dissolution rate, each of which contributes to the toxicological profile [15].

The aim of this review is to systematize current evidence on the behavior of NPs in the body in light of the factors listed above, to present a framework describing their sequential fate from administration to elimination, and to characterize the key biochemical mechanisms operating at each stage. The overall sequence of these stages is shown in Figure 1.

The clinical relevance of these stages is illustrated by the current landscape of approved and investigational nanomedicines, which extends well beyond oncology. Liposomal amphotericin B is used for systemic fungal infections, iron oxide nanoparticles for iron-deficiency anemia, lipid nanoparticles for hereditary transthyretin amyloidosis and, most recently, for mRNA vaccine delivery, illustrating that different disease contexts place emphasis on different stages of the NP life cycle—for example, deliberate MPS/reticuloendothelial uptake is exploited therapeutically for iron oxide formulations, whereas it is a barrier to be evaded for tumor-targeted carriers [16]. Clinical outcomes that diverge from preclinical expectations are similarly informative: BIND-014, a PSMA-targeted polymeric nanoparticle designed to enhance intratumoral docetaxel exposure, achieved a favorable and predictable pharmacokinetic profile in a first-in-human trial, yet objective responses remained modest across most tumor types, suggesting that active targeting and improved circulation kinetics alone do not guarantee proportional gains in efficacy and that later stages of the life cycle—tumor penetration, cellular uptake, and intracellular processing—can remain limiting even when earlier, systemic-level design goals are met [17].

## 2. Extracellular Stage

The route of administration establishes the initial conditions that shape the subsequent biological fate of NPs. Intravenous administration, which is standard for most therapeutic nanomedicines, results in complete and immediate entry of NPs into the systemic circulation. This produces a high initial concentration but also immediately exposes the particles to the complex biochemical environment of plasma [18]. Oral administration, by contrast, requires the particles to overcome multiple physiological barriers, including pronounced pH gradients within the gastrointestinal tract, enzymatic degradation, and the mucus barrier [19]. The latter consists of a dense layer of mucins—highly glycosylated proteins that form a hydrogel network capable of efficiently immobilizing and removing foreign particles [20]. Effective translocation through this barrier requires sufficient diffusional mobility, which can be achieved by modifying the NP surface with hydrophilic polymers such as polyethylene glycol. These polymers reduce nonspecific hydrophobic and electrostatic interactions with mucin [21]. Transepithelial transport in the intestine may potentially occur through the paracellular route; however, this pathway is severely restricted by tight junctions—specialized multiprotein structures that seal the intercellular spaces [22].

### 2.1. Protein Corona Formation and Its Role in Systemic Distribution

Regardless of the route of administration, entry of NPs into the systemic circulation triggers a key event of the extracellular stage: protein corona formation [23]. The high surface-area-to-volume ratio of NPs leads to the virtually instantaneous adsorption of plasma proteins, lipids, and metabolites, producing a dynamic multimolecular structure that defines the biological identity of the particle in vivo [20,24]. This process occurs within seconds and is not static. A distinction is made between the “soft” corona, composed of low-affinity proteins with high exchange rates, and the “hard” corona, formed by high-affinity proteins that bind almost irreversibly [20]. The hard corona is the principal long-term determinant of NP interactions with cells and tissues [24,25]. Corona composition depends on the physicochemical characteristics of the NPs—including size, shape, charge, hydrophobicity, and stiffness [26]—and on host-specific factors such as age, sex, and disease state [27]. Notably, corona composition depends not only on these intrinsic particle properties but also on how the effective particle dose is expressed: because protein adsorption is a surface-driven phenomenon, the ratio of particle surface area to protein concentration—rather than particle mass—determines which proteins are detected in the corona, and NP size itself alters corona composition through curvature effects independent of surface chemistry [28]. This dose-dependence of the corona has direct implications for the extent of cellular uptake and for organ-level distribution, discussed further below.

The pattern of protein corona formation directly determines the rate of NP uptake by tissue macrophages [29]. A key biochemical event is the adsorption of opsonins, including immunoglobulin G and complement components C3b and iC3b [30]. Receptors that recognize opsonized particles are expressed on cells of the mononuclear phagocyte system (MPS) and fall into two main groups: Fc gamma receptors (FcγRs), which bind the Fc region of immunoglobulins, and complement receptors (CRs), which recognize activated components of the complement cascade [31]. Their combined activation generates a strong phagocytic signal. Accordingly, the presence of opsonins on the NP surface signals MPS cells bearing the corresponding receptors: FcγRs recognize the Fc region of IgG, whereas CR1 binds C3b and C4b and CR3 and CR4 bind inactivated iC3b [31]. This initiates phagocytosis and rapid particle clearance [1]. Conversely, adsorption of so-called dysopsonins, including clusterin and albumin, may mask NPs from immune recognition and prolong their circulation time [24].

Shape and surface hydrophobicity likewise produce measurable, quantifiable differences in corona composition. Using mesoporous silica nanoparticles of identical chemistry, porosity, and surface potential, Madathiparambil Visalakshan et al. [32] showed that rod-shaped particles adsorbed substantially more total protein than spherical particles of matched dimensions, with immunoglobulins enriched on rods and albumin preferentially bound to spheres. Surface hydrophobicity produces a comparably specific shift in composition: across a continuous hydrophobicity gradient in gold NPs. Yu et al. [33] found that hydrophobic particles adsorbed 2.1-fold more total protein than hydrophilic counterparts, with serum albumin and fetal hemoglobin subunit beta increasing from 0.3% and 4% to 23% and 35% of the corona, respectively, as hydrophobicity increased, whereas vitronectin and antithrombin III preferentially bound the hydrophilic surfaces; apolipoproteins, by contrast, bound all particles irrespective of hydrophobicity.

Recent studies identify NP stiffness as a critical parameter that modulates corona composition. Li et al. [34] showed that NPs with intermediate Young’s moduli (75–700 kPa) preferentially adsorbed apolipoprotein A-I (ApoA1); notably, the relative abundance of ApoA1 was the only corona-protein parameter that correlated with NP circulation time. The authors proposed that this association may reflect reduced opsonization and altered receptor interactions, although the specific role of SR-B family receptors in this context remains to be clarified.

### 2.2. Systemic Clearance and the Role of the Mononuclear Phagocyte System

For most intravenously administered NPs, the principal sites of accumulation are the liver and spleen, where they are taken up by cells of the mononuclear phagocyte system (MPS), including Kupffer cells lining the hepatic sinusoids and macrophages in the splenic red pulp [35]. The efficiency of MPS-mediated uptake is determined by the extent of opsonization, particle size, and surface properties. NPs with a hydrodynamic diameter of less than 5.5 nm are generally able to evade MPS uptake and are rapidly eliminated by the kidneys through glomerular filtration [36].

NPs 10–100 nm in size exhibit the longest circulation times and may accumulate in certain target tissues through the passive mechanism known as the enhanced permeability and retention (EPR) effect [37]. This effect, which is the principal mechanism of passive targeting to solid tumors, arises from the abnormal architecture of the tumor endothelium, with its characteristic fenestrations, and from impaired lymphatic drainage, both of which promote extravasation and subsequent retention of NPs within the interstitial space [38,39]. However, the clinical relevance of the EPR effect is substantially limited: a systematic analysis of 117 publications showed that the median accumulation of NPs in solid tumors is only approximately 0.7% of the injected dose [40,41]. Importantly, this value varies by orders of magnitude depending on tumor type, animal model, and NP physicochemical properties, which casts doubt on the universality of passive EPR targeting and makes it a subject of ongoing debate.

This variability is well illustrated by two clinically approved nanomedicines of markedly different composition. PEGylated liposomal doxorubicin (Doxil/Caelyx) achieves a circulation half-life on the order of days and an area under the plasma concentration-time curve at least 60-fold greater than free doxorubicin, reflecting effective evasion of MPS uptake conferred by PEGylation [42]. By contrast, albumin-bound paclitaxel (nab-paclitaxel, Abraxane) is cleared far more rapidly, as the nanoparticle disassembles in circulation and paclitaxel redistributes onto endogenous albumin; its improved tumor delivery relative to solvent-based paclitaxel is instead attributed largely to gp60-mediated albumin transcytosis across tumor endothelium and possible SPARC binding, rather than to prolonged circulation [43]. This contrast illustrates that NP family and formulation chemistry, not particle size alone, determine the dominant mechanism and efficiency of tumor delivery.

This variance has been quantified systematically. A meta-analysis of murine biodistribution data compiled from 297 published studies found that tumor delivery efficiency differs significantly by cancer type, ranging from a median of 1.08% ID/g in ovarian tumor models to 3.10–4.22% ID/g in colon, liver, breast, and pancreatic models, and up to 6.18% ID/g in cervical tumor models. NP surface charge alone accounted for a significant fraction of this variability, with neutral NPs achieving nearly double the delivery efficiency of negatively charged NPs (4.00% vs. 2.34% ID/g) [44].

NPs larger than 200 nm are most efficiently taken up by splenic and hepatic macrophages and are rapidly removed from the circulation [45,46].

### 2.3. Transport Across Specialized Barriers

Access to specialized tissues such as the central nervous system requires NPs to cross additional barriers, particularly the blood–brain barrier (BBB). The BBB is formed by tight junctions between cerebral endothelial cells and is supported by pericytes and astrocytes [47]. This structure constitutes a highly selective barrier to most NPs. Transport across the BBB may occur by receptor-mediated transcytosis, for example through transferrin or low-density lipoprotein receptors, or by adsorptive-mediated transcytosis in the case of cationic NPs [48].

### 2.4. Interaction with Target Tissue and Cellular Internalization

After crossing the vascular barrier, NPs enter the interstitium, where they encounter the final extracellular obstacle: the extracellular matrix (ECM), composed of collagen, elastin, glycosaminoglycans, and fibronectin [49]. ECM density and architecture can substantially modulate NP diffusion. In desmoplastic tumors, in particular, excessive collagen deposition creates a pronounced steric barrier that limits deep tissue penetration by NPs and consequently reduces therapeutic efficacy [50]. Two strategies are being developed to overcome the steric barrier created by a dense ECM. The first involves coadministration of enzymes, such as collagenase or hyaluronidase, with the NPs or preinjection of these enzymes into the target tissue. The enzymes locally degrade matrix components, including collagen and hyaluronic acid, thereby temporarily increasing tissue permeability to subsequently administered NPs [51]. The second strategy focuses on designing NPs themselves with sizes and shapes favorable for diffusion, such as spherical particles 20–50 nm in diameter or nanorods with a high aspect ratio. Such particles can passively penetrate the interstitial space without additional enzymatic modulation [52].

To promote selective accumulation, NPs can be functionalized with high-affinity ligands, a strategy known as active targeting [53]. Its implementation nevertheless faces a fundamental limitation: the protein corona formed in vivo may shield ligands on the NP surface and prevent specific recognition by target receptors [54,55].

The extent of this masking is not fixed but depends on controllable design parameters. Ligands attached via short PEG linkers and presented at moderate-to-high surface density better withstand corona-mediated shielding and retain receptor binding, whereas long, flexible PEG spacers increase the likelihood that ligands become entangled within the adsorbed protein layer, reducing productive receptor engagement [56]. Consistent with this, cyclic RGD-functionalized nanoparticles retain receptor-binding capacity after corona formation even at low ligand density, although cellular uptake is still reduced by 26–76% relative to protein-free particles; short PEG linkers combined with moderate ligand density yield the best targeting performance [57]. These findings indicate that corona-mediated masking is a tunable rather than an inevitable obstacle, and that linker chemistry and ligand density are as important as ligand choice itself for preserving active-targeting function in vivo.

Monoclonal antibodies are among the most specific ligands; for example, trastuzumab can direct NPs to HER2, which is overexpressed in breast cancer [58]. However, antibody-based targeting entails high costs and potential immunogenicity. Peptide ligands such as RGD, which recognizes αvβ3 and αvβ5 integrins, provide a simpler and more economical alternative [59,60]. RGD-functionalized NPs have demonstrated high efficiency in delivering therapeutic agents to a broad range of tumor cells. Transferrin and its receptor constitute another well-studied pair for active targeting. Transferrin-conjugated NPs continue to be investigated in preclinical models [61]. MBP-426, a transferrin-conjugated liposomal formulation of oxaliplatin, underwent a phase I pharmacokinetic study in patients with advanced solid tumors [62].

## 3. Intracellular Stage

### 3.1. Mechanisms of NP Internalization

Interaction of protein-corona-coated NPs with the cell membrane initiates one or more internalization pathways. Clathrin-mediated endocytosis is the most common mechanism. Clathrin forms triskelion structures that assemble into a polyhedral lattice on the cytoplasmic side of the membrane. Following NP binding to a specific receptor, this lattice assembles with the participation of AP-2 adaptor proteins, which link clathrin to the receptors. The lattice stabilizes membrane invagination, which is then severed by the GTPase dynamin to form a clathrin-coated vesicle. Inside the cell, the vesicle loses its clathrin coat and fuses with early endosomes, directing the cargo toward lysosomal degradation [63].

In addition to clathrin-mediated endocytosis, NPs (particularly larger particles or those that induce pronounced actin remodeling at the plasma membrane) can be internalized via macropinocytosis. Unlike clathrin-mediated uptake, this actin-driven process does not depend on a specific cargo receptor: membrane ruffling engulfs extracellular fluid and associated particles in bulk, forming large, heterogeneous macropinosomes that subsequently join the endosomal system [64].

Caveolae-mediated endocytosis constitutes a second, clathrin-independent pathway based on flask-shaped plasma-membrane invaginations approximately 50–80 nm in diameter that are stabilized by caveolin and cavin proteins; this structural constraint makes the pathway particularly relevant for smaller NPs and for negatively charged or albumin-associated particles [64].

Because receptor engagement scales with the particle surface rather than with administered mass, particle size and surface area—not the injected mass—are the principal determinants of endocytic/phagocytic uptake rate across cell types and organs [65].

### 3.2. Endosome Formation and Maturation

After internalization, NP fate is determined by the dynamics of endosomal maturation, which ultimately govern the fate of both the particle and its payload. Once enclosed within endosomal vesicles, NPs encounter a progressively changing pH and enzymatic environment. Early endosomes (pH approximately 6.5) gradually mature into late endosomes (pH approximately 5.5) and then into endolysosomes (pH approximately 4.5) [66]. This acidification is driven by vacuolar H^+^-ATPase, an enzyme complex that transports protons into the vesicle. At the same time, marker proteins are exchanged: early endosomes lose the small GTPase Rab5 and acquire Rab7, a characteristic marker of late endosomes [66]. Upon fusion with lysosomes—organelles containing a broad range of acid hydrolases—NPs and their associated cargo can undergo acid- and enzyme-mediated degradation [67,68]. Organic NPs and biological macromolecules may be completely degraded.

The entry pathway engaged during internalization also shapes this maturation process and its outcome. Cargo internalized via clathrin-coated vesicles is efficiently funneled into the classical early-to-late endosome-to-lysosome route. By contrast, macropinosomes and caveolar vesicles are, in several cell types, less tightly coupled to this route: their content has been reported to more readily escape complete lysosomal degradation, in some cases bypassing the endosome-to-lysosome route entirely and trafficking instead to caveosomes or directly to the endoplasmic reticulum/Golgi—although this behavior remains cell-type-dependent and is debated, as caveolar cargo has also been shown to be redirected to late endosomes/lysosomes in other systems [64,69]. Because pathway selection therefore directly influences whether an NP payload is degraded or delivered intact to the cytoplasm, the balance between clathrin-, caveolae-, and macropinocytosis-dependent uptake is a key design consideration for lysosome-sensitive therapeutics.

For many therapeutic strategies, particularly nucleic acid delivery, lysosomal degradation is a major limitation and necessitates the development of approaches for endosomal escape [70].

### 3.3. Intracellular Delivery and Effects of NPs

Delivery to subcellular compartments. Gene delivery and the delivery of certain cytostatic agents require access to the nucleus. Small NPs (<5 nm) may passively diffuse through nuclear pore complexes [71]. Larger particles require active transport mediated by nuclear localization signal (NLS) peptides. To deliver genetic material or cytostatic agents to the nucleus, NPs are commonly conjugated to NLS peptides—clusters of positively charged amino acids [72]. These peptides are recognized by the importin α/β complex, which transports the NPs through the nuclear pore. In the nucleoplasm, the high concentration of Ran·GTP induces complex dissociation and releases the NPs into the nucleus, after which the importins are recycled [73]. This approach is widely used to deliver nucleic acids and agents that act at the transcriptional level.

NPs can markedly affect cellular energy metabolism, primarily through mitochondrial effects [2]. For targeted modulation of mitochondrial function, nanocarriers can be functionalized with lipophilic triphenylphosphonium (TPP^+^) cations or TPP^+^-containing moieties [74,75]. Driven by the negative potential across the inner mitochondrial membrane, these cations preferentially accumulate within mitochondria and can facilitate the delivery of antioxidants or anticancer agents [76]. Beyond targeted delivery, NPs can affect energy metabolism directly by interacting with components of the respiratory chain. Several mechanisms of such direct action have been described. NPs may bind to iron–sulfur clusters or hemes within respiratory-chain complexes, thereby disrupting electron flow. Costa et al. [77] reported that silver NPs at concentrations of 10–50 mg/L inhibited the activity of all four respiratory-chain complexes in mitochondria isolated from rat brain, skeletal muscle, heart, and liver. Some NPs, particularly cationic and amphiphilic particles, disrupt the integrity of the inner mitochondrial membrane, rendering it permeable to protons [78,79]. This uncouples respiration from ATP synthesis: electrons continue to be transported along the chain, but the energy of the proton gradient is dissipated as heat rather than used for ADP phosphorylation. Many NPs, especially those based on transition metals, and quantum dots generate reactive oxygen species (ROS) that damage mitochondrial membrane lipids, respiratory-chain proteins, and mitochondrial DNA, causing mitochondrial dysfunction and reduced ATP production and potentially triggering apoptosis through the mitochondrial pathway [80]. This mechanism underlies a therapeutic strategy for the selective suppression of energy metabolism in cancer cells, which is regarded as a promising approach in oncology [81]. At the same time, some NPs can exert the opposite effect and increase ATP production. Hosseini et al. [82] observed increased glucose-stimulated ATP production in pancreatic islets pretreated with CeO_2_ and Y_2_O_3_ NPs. This effect is probably related to protection of the cells against oxidative stress rather than to direct stimulation of the respiratory chain.

NPs can modulate cellular signaling and the immune response, and these effects are often mediated by the same mechanisms that underlie their toxicity. Reactive oxygen species generated during NP–mitochondria interactions activate the transcription factor nuclear factor κB (NF-κB) [83,84]. The mechanism involves phosphorylation of the inhibitory protein IκB by IKK kinases, its ubiquitin-dependent degradation, and subsequent translocation of NF-κB to the nucleus, where it stimulates transcription of the proinflammatory cytokines TNF-α, IL-1β, and IL-6 [85]. In this way, silica and titanium dioxide NPs promote synthesis of pro-IL-1β, providing the first, “priming,” signal required for subsequent activation of the NOD-like receptor family pyrin domain-containing 3 (NLRP3) inflammasome [86]. The same nanomaterials provide the second signal by activating the multiprotein cytosolic NLRP3 complex, which recognizes pathogen-associated and damage-associated molecular patterns [87]. Inflammasome activation comprises two stages: priming, involving pro-IL-1β synthesis, and activation itself, triggered by NP phagocytosis, ROS generation, or lysosomal damage. The activated NLRP3 sensor oligomerizes and recruits the ASC adaptor protein, which activates procaspase-1. Mature caspase-1 cleaves pro-IL-1β and pro-IL-18, enabling secretion of their mature forms and eliciting a pronounced inflammatory response [86].

The overall decision process governing nanoparticle intracellular fate from the route of internalization to endosomal escape or lysosomal degradation and the resulting nuclear or mitochondrial effects is summarized in Figure 2.

## 4. Elimination

Removal of NPs from the body comprises two sequential stages: intracellular degradation followed by the release of degradation products or intact particles from the cell. This size- and opsonization-dependent selection between the renal and hepatobiliary elimination routes is summarized in Figure 3.

As noted above, both corona composition and the rate of cellular uptake depend on the particle surface presented per unit dose rather than on administered mass. This has direct consequences for elimination as well: because Kupffer cell phagocytic capacity is saturable, hepatic clearance efficiency depends on the number of NPs delivered per unit time rather than on the injected mass. Ouyang et al. demonstrated a dose threshold of approximately 10^12^ NPs per mouse, above which Kupffer cell uptake becomes saturated, nonlinearly reducing hepatic clearance and correspondingly prolonging circulation and increasing extrahepatic accumulation; this threshold behavior was consistent across gold, silica, and liposomal nanomaterials [96]. Together, these findings indicate that particle number and surface area, rather than mass, are the operative dose metrics governing corona formation, cellular uptake, and the efficiency and organ distribution of NP elimination.

### 4.1. Intracellular Degradation of NPs

For inorganic NPs, the key process is dissolution in the acidic lysosomal environment, accompanied by the release of metal ions. Zinc oxide and iron oxide NPs, for example, readily dissolve and release Zn^2+^ and Fe^2+^/Fe^3+^ [97]. The released iron ions may enter metabolic pathways through binding to ferritin and transferrin, thereby reducing the long-term toxicity of biodegradable magnetic NPs relative to nonbiodegradable analogues [98]. However, excess free iron catalyzes the Fenton reaction and induces local oxidative stress [92]. Organic NPs, such as those based on poly(lactic-co-glycolic acid), undergo predominantly nonenzymatic hydrolysis of ester bonds [97].

### 4.2. Renal Clearance

NPs are eliminated from the body primarily through the kidneys and liver, with the predominant route determined by the combined physicochemical properties of the particles [99,100]. Renal clearance is particularly important for small NPs and is mediated by the glomerular filtration barrier.

The glomerular filtration barrier is a three-layered structure comprising fenestrated capillary endothelium with a negatively charged glycocalyx; a 300–350-nm-thick glomerular basement membrane composed of type IV collagen, laminin, nidogen, and proteoglycans; and podocytes whose interdigitating foot processes form 4–11-nm filtration slits closed by slit diaphragms [88,89]. Together, these layers create a selectivity threshold based on size and charge: particles with a hydrodynamic diameter of less than 6 nm are filtered and excreted in urine, whereas particles larger than 8 nm are generally retained [90]. This threshold, however, holds for rigid spherical particles in the absence of a protein corona; protein adsorption increases the effective diameter, while charge and deformability further modulate filterability [91,101,102].

### 4.3. Hepatobiliary Clearance

For NPs that exceed the renal filtration threshold, hepatobiliary clearance is the principal route of elimination. Large opsonized NPs are phagocytosed by Kupffer cells and by tissue macrophages in other MPS organs [92,103]. Smaller particles (less than 100–150 nm) may pass through endothelial fenestrae into the space of Disse and reach hepatocytes, from which either the particles themselves or their biotransformation products are excreted into bile and eliminated in feces [92]. The rate and efficiency of this process depend on particle size, surface properties, and biodegradability. The biliary excretion of soluble degradation products and metabolites may involve canalicular ABC transporters, including MRP2 and BCRP [93,94,95]. Nonbiodegradable NPs may persist in the liver and spleen for many months, raising concerns about chronic organ toxicity [92]. By contrast, biodegradable iron oxide NPs are degraded in lysosomes, and the released iron can be reutilized through incorporation into ferritin, hemoglobin, and other iron-containing proteins [92].

## 5. Conclusions

This review demonstrates that nanoparticle (NP) behavior in the body is an integrated, multistep process governed by a dynamic interplay between nanoparticle physicochemical properties and the biological environment. Each stage starting from protein corona formation to cellular uptake, intracellular trafficking, systemic distribution, and elimination can modify NP properties and thereby determine its fate at subsequent levels of organization, rather than operating as an independent event.

An important implication of this integrated view is that nanoparticle safety and efficacy cannot be predicted from a single physicochemical parameter or from isolated cellular assays; NP behavior should instead be evaluated as a continuum spanning the molecular, cellular, tissue, and systemic levels. This perspective also highlights the need for standardized experimental frameworks that enable meaningful comparison among nanoparticle platforms. In particular, reporting only mass-based doses may obscure major differences in particle number, surface area, and biological reactivity between formulations; greater harmonization of dosing metrics, characterization procedures, corona analysis, biodistribution measurements, and toxicity endpoints would substantially improve the reproducibility and translational relevance of nanomedicine research.

Several areas, while already under active investigation, have yet to yield validated, standardized solutions and remain priorities for future work. Physiologically based pharmacokinetic and pharmacodynamic models incorporating nanoparticle-specific parameters are advancing rapidly, but broad incorporation of sex-, age-, genetic-, and disease-related variability remains limited [5,65]. Complementary dose metrics (including particle number and surface area alongside mass) are increasingly used in individual studies, yet their routine, field-wide adoption is still lacking [96]. Multi-omics approaches (proteomics, lipidomics, metabolomics, transcriptomics) are being actively integrated with nanoparticle research [104], though standardized workflows linking these data to biodistribution and toxicity endpoints are still emerging. Similarly, the temporal evolution of the protein corona during circulation, cellular uptake, and tissue accumulation is receiving growing attention [28], but a predictive, generalizable model of this dynamic process remains unavailable. Next-generation nanomedicine may further benefit from adaptive, biologically responsive nanosystems whose surface composition, charge, or structural properties change in response to the patient-specific microenvironment, enabling controlled modulation of the corona, tissue-specific targeting, and reduced off-target accumulation.

Overall, the future development of nanomedicines will require a shift from viewing nanoparticles as static drug carriers toward understanding them as dynamic biological entities whose identity and function continuously evolve after administration. A mechanistic understanding of the nanoparticle–biological interface, combined with standardized characterization, predictive modeling, and adaptive nanomaterial design, will be essential for improving the safety, efficacy, reproducibility, and clinical translation of nanoparticle-based therapeutics.

## Figures and Tables

**Figure 1:**
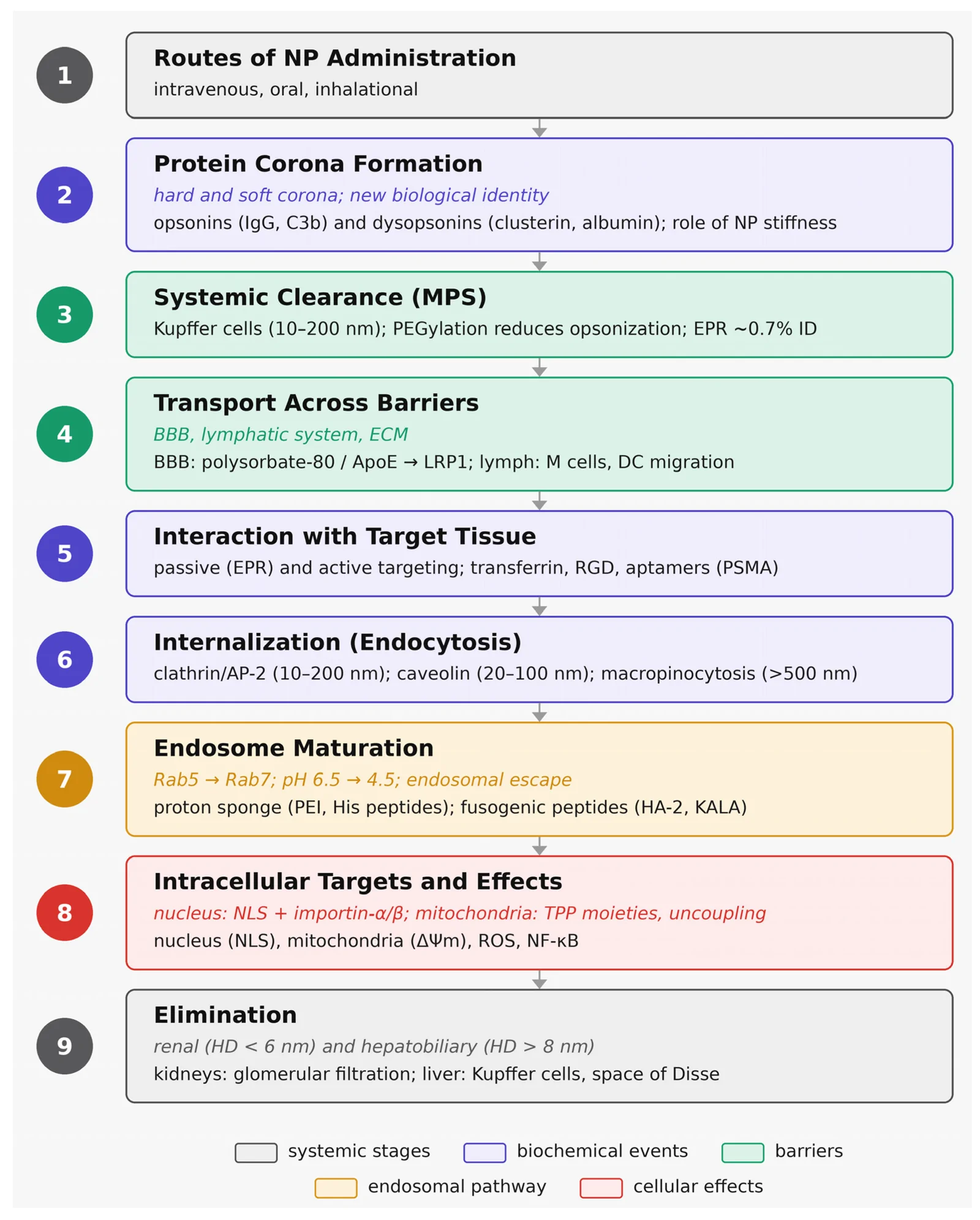
Integrated schematic of the “life cycle” of nanoparticles in the body. AP-2, adaptor protein 2; ApoE, apolipoprotein E; BBB, blood–brain barrier; C3b, complement component 3b; DC, dendritic cell; ECM, extracellular matrix; EPR, enhanced permeability and retention; HA-2, influenza haemagglutinin-2 peptide; HD, hydrodynamic diameter; ID, injected dose; IgG, immunoglobulin G; LRP1, low-density lipoprotein receptor-related protein 1; MPS, mononuclear phagocyte system; NF-κB, nuclear factor κB; NLS, nuclear localization signal; NP, nanoparticle; PEG, polyethylene glycol; PEI, polyethylenimine; PSMA, prostate-specific membrane antigen; RGD, arginine–glycine–aspartate motif; ROS, reactive oxygen species; TPP, triphenylphosphonium; ΔΨm, mitochondrial membrane potential.

**Figure 2:**
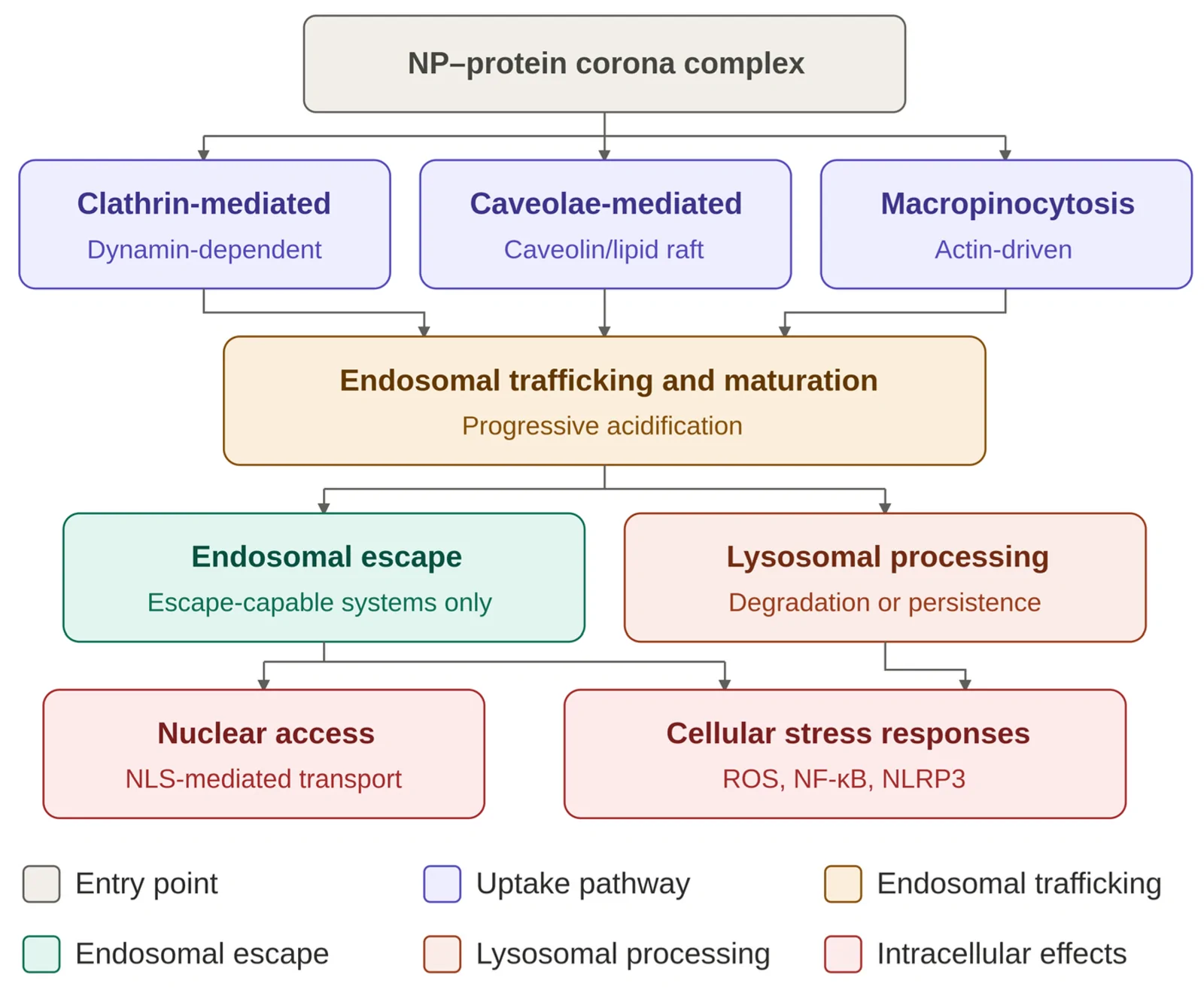
Decision map of nanoparticle intracellular fate. Following formation of a protein corona (i.e., a dynamic layer that continues to evolve after cellular entry) NPs are internalized via clathrin-mediated endocytosis, caveolae-mediated endocytosis, or macropinocytosis, depending on particle size, surface charge, and membrane interactions [63]. Internalized cargo undergoes endosomal trafficking and maturation (substitution of Rab5 to Rab7, progressive acidification) before one of two outcomes: endosomal escape, which is not the default fate but requires escape-capable nanocarrier design, or lysosomal processing, which may result in degradation, dissolution, or persistence depending on NP composition [66,67,68,70]. Both outcomes can trigger cellular stress responses (ROS generation, NF-κB signaling, NLRP3 inflammasome activation), as these can arise from cytosolic delivery as well as from lysosomal membrane damage [83,84,85,86,87]. Nuclear access following endosomal escape occurs via NLS-mediated active transport through the importin α/β complex; passive diffusion through the nuclear pore complex is restricted to sufficiently small species [71,72,73].

**Figure 3:**
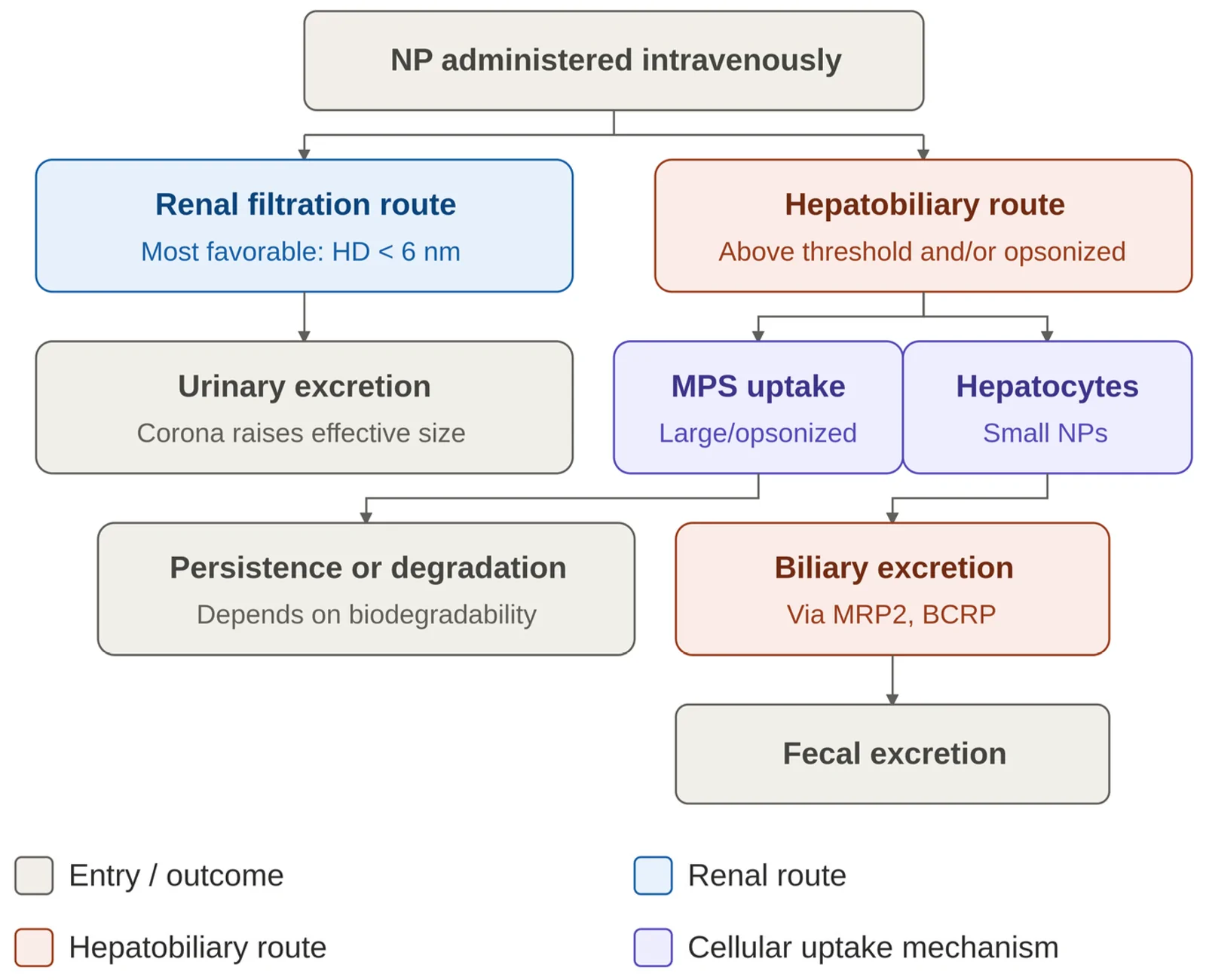
Size- and opsonization-dependent selection between renal and hepatobiliary elimination routes. Renal filtration is most favorable for NPs with a hydrodynamic diameter below approximately 6 nm; protein corona formation increases the effective particle diameter and can shift particles above this range [88,89,90,91]. NPs above the renal filtration threshold and/or bearing an opsonized surface undergo hepatobiliary clearance. Large, opsonized particles are predominantly taken up by the mononuclear phagocyte system (MPS) (chiefly Kupffer cells and splenic macrophages) where their subsequent fate (long-term tissue persistence versus lysosomal degradation) depends on NP biodegradability [92]. Smaller particles instead reach hepatocytes via endothelial fenestrae and the space of Disse, undergo biliary excretion mediated by canalicular ABC transporters including MRP2 and BCRP, and are eliminated in feces [92,93,94,95].

## Data Availability

No new data were created or analyzed in this study. Data sharing is not applicable to this article.

## Abbreviations

The following abbreviations are used in this manuscript:

ABC	ATP-binding cassette
ApoA1	Apolipoprotein A-I
BBB	Blood–brain barrier
ECM	Extracellular matrix
EPR	Enhanced permeability and retention
HD	Hydrodynamic diameter
MPS	Mononuclear phagocyte system
NF-κB	Nuclear factor κB
NLRP3	NOD-like receptor family pyrin domain-containing 3
NLS	Nuclear localization signal
NP	Nanoparticle
PBPK	Physiologically based pharmacokinetic
PEG	Polyethylene glycol
ROS	Reactive oxygen species
TPP	Triphenylphosphonium

## References

[1] Zhang Y., Bai Y., Jia J., Gao N., Li Y., Zhang R., Jiang G., Yan B. (2014). Perturbation of physiological systems by nanoparticles. Chem. Soc. Rev..

[2] Gomes A., Sengupta J., Datta P., Ghosh S., Gomes A. (2016). Physiological interactions of nanoparticles in energy metabolism, immune function and their biosafety: A review. J. Nanosci. Nanotechnol..

[3] Mazayen Z.M., Ghoneim A.M., Elbatanony R.S., Basalious E.B., Bendas E.R. (2022). Pharmaceutical nanotechnology: From the bench to the market. Future J. Pharm. Sci..

[4] Chang J., Yu B., Saltzman W.M., Girardi M. (2023). Nanoparticles as a Therapeutic Delivery System for Skin Cancer Prevention and Treatment. JID Innov..

[5] Peralta-Cuevas E., Degollado-Hernández N.Y., Martínez-Ortiz I.C., Gutierrez-Onofre A.J., Garcia-Atutxa I., Villanueva-Flores F. (2025). How Do Nanoparticle Properties Shape Pharmacokinetics and Pharmacodynamics? A Mechanistic Review. Front. Pharmacol..

[6] Farokhzad O.C., Langer R. (2009). Impact of nanotechnology on drug delivery. ACS Nano.

[7] Haripriyaa M., Suthindhiran K. (2023). Pharmacokinetics of nanoparticles: Current knowledge, future directions and its implications in drug delivery. Future J. Pharm. Sci..

[8] Hagens W.I., Oomen A.G., de Jong W.H., Cassee F.R., Sips A.J.A.M. (2007). What do we (need to) know about the kinetic properties of nanoparticles in the body?. Regul. Toxicol. Pharmacol..

[9] Li M., Al-Jamal K.T., Kostarelos K., Reineke J. (2010). Physiologically based pharmacokinetic modeling of nanoparticles. ACS Nano.

[10] Chou W.-C., Lin Z. (2024). Impact of protein coronas on nanoparticle interactions with tissues and targeted delivery. Curr. Opin. Biotechnol..

[11] Tekie F.S.M., Hajiramezanali M., Geramifar P., Raoufi M., Dinarvand R., Soleimani M., Atyabi F. (2020). Controlling evolution of protein corona: A prosperous approach to improve chitosan-based nanoparticle biodistribution and half-life. Sci. Rep..

[12] Pustulka S.M., Ling K., Pish S.L., Champion J.A. (2020). Protein Nanoparticle Charge and Hydrophobicity Govern Protein Corona and Macrophage Uptake. ACS Appl. Mater. Interfaces.

[13] Ashkarran A.A., Gharibi H., Grunberger J.W., Saei A.A., Khurana N., Mohammadpour R., Ghandehari H., Mahmoudi M. (2023). Sex-Specific Silica Nanoparticle Protein Corona Compositions Exposed to Male and Female BALB/c Mice Plasmas. ACS Bio Med. Chem. Au.

[14] Zelepukin I.V., Shevchenko K.G., Deyev S.M. (2024). Rediscovery of mononuclear phagocyte system blockade for nanoparticle drug delivery. Nat. Commun..

[15] Abbasi R., Shineh G., Mobaraki M., Doughty S., Tayebi L. (2023). Structural parameters of nanoparticles affecting their toxicity for biomedical applications: A review. J. Nanopart. Res..

[16] Anselmo A.C., Mitragotri S. (2019). Nanoparticles in the clinic: An update. Bioeng. Transl. Med..

[17] Von Hoff D.D., Mita M.M., Ramanathan R.K., Weiss G.J., Mita A.C., LoRusso P.M., Burris H.A., Hart L.L., Low S.C., Parsons D.M. (2016). Phase I Study of PSMA-Targeted Docetaxel-Containing Nanoparticle BIND-014 in Patients with Advanced Solid Tumors. Clin. Cancer Res..

[18] Lasa-Saracíbar B., El Moukhtari S.H., Tsotakos T., Xanthopoulos S., Loudos G., Bouziotis P., Blanco-Prieto M.J. (2022). In vivo biodistribution of edelfosine-loaded lipid nanoparticles radiolabeled with Technetium-99m: Comparison of administration routes in mice. Eur. J. Pharm. Biopharm..

[19] Zhao C., Wen S., Xu R., Wang K., Zhong Y., Huang D., Zhao B., Chen W. (2025). Oral delivery of ultra-small zwitterionic nanoparticles to overcome mucus and epithelial barriers for macrophage modulation and colitis therapy. Acta Biomater..

[20] Yusuf A., Almotairy A.R.Z., Henidi H., Alshehri O.Y., Aldughaim M.S. (2023). Nanoparticles as Drug Delivery Systems: A Review of the Implication of Nanoparticles’ Physicochemical Properties on Responses in Biological Systems. Polymers.

[21] Suk J.S., Xu Q., Kim N., Hanes J., Ensign L.M. (2016). PEGylation as a strategy for improving nanoparticle-based drug and gene delivery. Adv. Drug Deliv. Rev..

[22] Ramirez-Velez I., Namjoshi A.A., Effiong U.M., Peppas N.A., Belardi B. (2024). Paracellular Delivery of Protein Drugs with Smart EnteroPatho Nanoparticles. ACS Nano.

[23] García-Álvarez R., Hadjidemetriou M., Sánchez-Iglesias A., Liz-Marzán L.M., Kostarelos K. (2018). In vivo formation of protein corona on gold nanoparticles. The effect of their size and shape. Nanoscale.

[24] Monopoli M.P., Åberg C., Salvati A., Dawson K.A. (2012). Biomolecular coronas provide the biological identity of nanosized materials. Nat. Nanotechnol..

[25] Zanganeh M., Mason T.O., Eskandari H., Rupreo V., Bagheri F., Farzadfard A., Shojaosadati S.A., Morshedi D., Sutherland D.S., Buell A.K. (2026). Shear-sensitive clustering of nanoparticles and their protein corona composition govern α-synuclein aggregation. Environ. Sci. Nano.

[26] Bewersdorff T. (2022). Systematic Investigations on the Protein Corona of Different Nanocarriers and Its Role in Cellular Uptake. Ph.D. Thesis.

[27] Cisneros E.P., Morse B.A., Savk A., Malik K., Peppas N.A., Lanier O.L. (2024). The role of patient-specific variables in protein corona formation and therapeutic efficacy in nanomedicine. J. Nanobiotechnol..

[28] Lundqvist M., Augustsson C., Lilja M., Lundkvist K., Dahlbäck B., Linse S., Cedervall T. (2017). The nanoparticle protein corona formed in human blood or human blood fractions. PLoS ONE.

[29] Lins A., Fortmann M., Mulac-Hahnen D., Humpf H.-U., Langer K. (2026). The nanoparticle protein corona impacts interactions with monocytes and macrophages. Int. J. Pharm..

[30] Qiu Y., He T., Miao C., Shi X., Niu Y., Mailänder V., Crespy D., Landfester K., Jiang S. (2026). The Distribution of Complement Proteins in Soft and Hard Coronas Impacts Macrophage Uptake of Nanoparticles. Adv. Healthc. Mater..

[31] Peiser L. (2016). Phagocytosis: Enhancement. Encyclopedia of Life Sciences.

[32] Madathiparambil Visalakshan R., González García L.E., Benzigar M.R., Ghazaryan A., Simon J., Mierczynska-Vasilev A., Michl T.D., Vinu A., Mailänder V., Morsbach S. (2020). The Influence of Nanoparticle Shape on Protein Corona Formation. Small.

[33] Yu Q., Zhao L., Guo C., Yan B., Su G. (2020). Regulating Protein Corona Formation and Dynamic Protein Exchange by Controlling Nanoparticle Hydrophobicity. Front. Bioeng. Biotechnol..

[34] Li M., Jin X., Liu T., Fan F., Gao F., Chai S., Yang L. (2022). Nanoparticle elasticity affects systemic circulation lifetime by modulating adsorption of apolipoprotein A-I in corona formation. Nat. Commun..

[35] Lee A.-R., Nam K., Lee B.J., Lee S.-W., Baek S.-M., Bang J.-S., Choi S.-K., Park S.-J., Kim T.-H., Jeong K.-S. (2019). Hepatic Cellular Distribution of Silica Nanoparticles by Surface Energy Modification. Int. J. Mol. Sci..

[36] Choi H.S., Liu W., Misra P., Tanaka E., Zimmer J.P., Itty Ipe B., Bawendi M.G., Frangioni J.V. (2007). Renal clearance of quantum dots. Nat. Biotechnol..

[37] Aldayel A.M., Hufnagel S., O’Mary H.L., Valdes S.A., Alzhrani R.F., Xu H., Cui Z. (2023). Effect of nanoparticle size on their distribution and retention in chronic inflammation sites. Discov. Nano.

[38] Matsumura Y., Maeda H. (1986). A new concept for macromolecular therapeutics in cancer chemotherapy: Mechanism of tumoritropic accumulation of proteins and the antitumor agent SMANCS. Cancer Res..

[39] Nguyen L.N.M., Ngo W., Lin Z.P., Sindhwani S., MacMillan P., Mladjenovic S.M., Chan W.C.W. (2024). The mechanisms of nanoparticle delivery to solid tumours. Nat. Rev. Bioeng..

[40] Wilhelm S., Tavares A.J., Dai Q., Ohta S., Audet J., Dvorak H.F., Chan W.C.W. (2016). Analysis of nanoparticle delivery to tumours. Nat. Rev. Mater..

[41] Shi J., Kantoff P.W., Wooster R., Farokhzad O.C. (2017). Cancer nanomedicine: Progress, challenges and opportunities. Nat. Rev. Cancer.

[42] Gabizon A., Shmeeda H., Barenholz Y. (2003). Pharmacokinetics of Pegylated Liposomal Doxorubicin: Review of Animal and Human Studies. Clin. Pharmacokinet..

[43] Yardley D.A. (2013). nab-Paclitaxel mechanisms of action and delivery. J. Control. Release.

[44] Chen Q., Yuan L., Chou W.-C., Cheng Y.-H., He C., Monteiro-Riviere N.A., Riviere J.E., Lin Z. (2023). Meta-Analysis of Nanoparticle Distribution in Tumors and Major Organs in Tumor-Bearing Mice. ACS Nano.

[45] Cho M., Cho W.S., Choi M., Kim S.J., Han B.S., Kim S.H., Kim H.O., Sheen Y.Y., Jeong J. (2009). The impact of size on tissue distribution and elimination by single intravenous injection of silica nanoparticles. Toxicol. Lett..

[46] Nowak-Jary J., Machnicka B. (2022). Pharmacokinetics of magnetic iron oxide nanoparticles for medical applications. J. Nanobiotechnol..

[47] He Y., Qu M., Yu L., He L., Lu Y., Hong J., Sun M., Yang H., Mi W., Guo H. (2026). Blood–Brain Barrier: Structure, Function, Diseases, and Drug Delivery Systems. MedComm.

[48] Sarkar A.K., Jana S., Kaur G., Arora H., Barman S., Mukherjee N., Shaw S., Maity A.R., Seth P., Jana N.R. (2025). Chemically Designed Functional Nanomicelles for Blood-Brain Barrier Crossing. Nano Lett..

[49] Theocharis A.D., Skandalis S.S., Gialeli C., Karamanos N.K. (2016). Extracellular matrix structure. Adv. Drug Deliv. Rev..

[50] Hwang J.-E., Jeon M., Yim H., Park J., Kim H.-S., Lee S., Park H., Lee J.K., Im H.-J. (2026). Collagenase-Functionalized Liposomes Overcome Stromal Barriers in Pancreatic Cancer. ACS Nano.

[51] Shin S.-H., Viswanath D., Harper H.A., Torregrosa-Allen S.E., McMahan C.J., Schwimmer A.E., Elzey B.D., Won Y.-Y. (2026). Enhancing intratumoral spread of radioluminescent nanoparticles via collagenase functionalization for radiation-induced photodynamic cancer therapy. Nanomed. Nanotechnol. Biol. Med..

[52] Lee J., Zhang W., Nguyen D., Zhou L., Amengual J., Zhai J., Cote T., Landolina M., Ahmadi E., Sands I. (2025). Computation-aided design of rod-shaped nanoparticles for tumoral targeting. J. Control. Release.

[53] Noh K., Yi S., Kim H., Lee J., Kim S., Yoo W., Jung E., Choi J., Park H., Hwang S. (2025). Targeting CD155 in lung adenocarcinoma: A5 nanobody-based therapeutics for precision treatment and enhanced drug delivery. Signal Transduct. Target. Ther..

[54] Li J., Yu J., Song J., Zhang Y., Li N., Wang Z., Qin M., Zhao M., Zhang B., Huang R. (2025). Galloylated liposomes enable targeted drug delivery by overcoming protein corona shielding. Nat. Commun..

[55] Zhang T., Li J., Lu J., Li J., Zhang H., Miao Y., Liu X., He Y., Yang L., Fan H. (2025). Enhanced tumor-targeting ability of transferrin-functionalized magnetic nanoparticles by in vivo AMF stimulation. Biomaterials.

[56] Abstiens K., Gregoritza M., Goepferich A.M. (2019). Ligand Density and Linker Length are Critical Factors for Multivalent Nanoparticle–Receptor Interactions. ACS Appl. Mater. Interfaces.

[57] Su G., Jiang H., Xu B., Yu Y., Chen X. (2018). Effects of Protein Corona on Active and Passive Targeting of Cyclic RGD Peptide-Functionalized PEGylation Nanoparticles. Mol. Pharm..

[58] Hughes C.S., Tayeb S., Muir D., Hayes A.J., Watson P., Jones A.T. (2026). Rapid plasma membrane reorganisation and endocytosis in HER2 breast cancer cells incubated with trastuzumab decorated polymer nanoparticles. RSC Pharm..

[59] Kapp T.G., Rechenmacher F., Neubauer S., Maltsev O.V., Cavalcanti-Adam E.A., Zarka R., Reuning U., Notni J., Wester H.-J., Mas-Moruno C. (2017). A Comprehensive Evaluation of the Activity and Selectivity Profile of Ligands for RGD-binding Integrins. Sci. Rep..

[60] Jackson N., Bouzeineddine N., Cecchi D., Holder S., Gee K., Beckham W., Basta S., Krishnan S., Chithrani D.B. (2025). Comprehensive analysis of the tumor targeting efficiency of functionalized nanoparticles in an immunocompetent environment. Sci. Rep..

[61] Rong W., Zhang M., Yang S., Wang K., Yang X., Zhang G., Li Z., Ding Y., Zhang T., Gai Y. (2026). Gambogic acid-loaded transferrin-modified liposomes enhancing potent ferroptosis in breast cancer. Sci. Rep..

[62] Sankhala K.K., Mita A.C., Adinin R., Wood L., Beeram M., Bullock S., Yamagata N., Matsuno K., Fujisawa T., Phan A. (2009). A phase I pharmacokinetic (PK) study of MBP-426, a novel liposome encapsulated oxaliplatin. J. Clin. Oncol..

[63] Kaksonen M., Roux A. (2018). Mechanisms of clathrin-mediated endocytosis. Nat. Rev. Mol. Cell Biol..

[64] Manzanares D., Ceña V. (2020). Endocytosis: The Nanoparticle and Submicron Nanocompounds Gateway into the Cell. Pharmaceutics.

[65] Chou W.-C., Cheng Y.-H., Riviere J.E., Monteiro-Riviere N.A., Kreyling W.G., Lin Z. (2022). Development of a multi-route physiologically based pharmacokinetic (PBPK) model for nanomaterials: A comparison between a traditional versus a new route-specific approach using gold nanoparticles in rats. Part. Fibre Toxicol..

[66] Huotari J., Helenius A. (2011). Endosome maturation. EMBO J..

[67] Lee D., Hong J.H. (2020). Nanoparticle-Mediated Therapeutic Application for Modulation of Lysosomal Ion Channels and Functions. Pharmaceutics.

[68] Wu M., Zhang M., Zhang Y., Li Z., Li X., Liu Z., Liu H., Li X. (2021). Relationship between lysosomal dyshomeostasis and progression of diabetic kidney disease. Cell Death Dis..

[69] Qiu C., Xia F., Zhang J., Shi Q., Meng Y., Wang C., Pang H., Gu L., Xu C., Guo Q. (2023). Advanced strategies for overcoming endosomal/lysosomal barrier in nanodrug delivery. Research.

[70] Johansson J.M., Du Rietz H., Hedlund H., Eriksson H.C., Oude Blenke E., Pote A., Harun S., Nordenfelt P., Lindfors L., Wittrup A. (2025). Cellular and biophysical barriers to lipid nanoparticle mediated delivery of RNA to the cytosol. Nat. Commun..

[71] Ma X., Gong N., Zhong L., Sun J., Liang X.-J. (2016). Future of nanotherapeutics: Targeting the cellular sub-organelles. Biomaterials.

[72] Li N., Yang H., Yu Z., Li Y., Pan W., Wang H., Tang B. (2017). Nuclear-targeted siRNA delivery for long-term gene silencing. Chem. Sci..

[73] Geraldo M.T., Takeda A.A.S., Braz A.S.K., Lemke N. (2016). Bending-Twisting Motions and Main Interactions in Nucleoplasmin Nuclear Import. PLoS ONE.

[74] Boddapati S.V., D’Souza G.G.M., Erdogan S., Torchilin V.P., Weissig V. (2008). Organelle-targeted nanocarriers: Specific delivery of liposomal ceramide to mitochondria enhances its cytotoxicity in vitro and in vivo. Nano Lett..

[75] Kaneko M., Yamazaki H., Ono T., Horie M., Ito A. (2023). Effective magnetic hyperthermia induced by mitochondria-targeted nanoparticles modified with triphenylphosphonium-containing phospholipid polymers. Cancer Sci..

[76] Pashirova T.N., Nemtarev A.V., Souto E.B., Mironov V.F. (2023). Triarylphosphonium compounds as effective vectors for mitochondria-targeted delivery systems: Decoration strategies and prospects for clinical application. Russ. Chem. Rev..

[77] Costa C.S., Ronconi J.V.V., Daufenbach J.F., Gonçalves C.L., Rezin G.T., Streck E.L., da Silva Paula M.M. (2010). In vitro effects of silver nanoparticles on the mitochondrial respiratory chain. Mol. Cell. Biochem..

[78] Bhattacharjee S., Ershov D., Islam M.A., Kämpfer A.M., Maslowska K.A., van der Gucht J., Alink G.M., Marcelis A.T.M., Zuilhof H., Rietjens I.M.C.M. (2014). Role of membrane disturbance and oxidative stress in the mode of action underlying the toxicity of differently charged polystyrene nanoparticles. RSC Adv..

[79] Wang T., Deng Y., Chen Y., Qu G., Feng Z., Shang J., Zheng J., He N. (2019). Disordered Metabolism and Repair Mechanism: Mitochondria Influenced by Cationic and Neutral Nanoparticles. J. Biomed. Nanotechnol..

[80] Mohamed H.R.H., Mohamed B.A., Hakeem G.M., Elnawasani S.H., Nagy M., Essam R., Diab A., Safwat G. (2025). Cobalt oxide nanoparticles induce cytotoxicity and excessive ROS mediated mitochondrial dysfunction and p53-independent apoptosis in melanoma cells. Sci. Rep..

[81] Xu X., Huang Q., Liu Y., Liu J., Yang D., Song Y., Han X. (2026). Smart Nanoparticles Disrupting Energy Supply through Triple Mechanisms to Kill Tumors via Dual Disruption of Mitochondria and Lysosomes. Adv. Sci..

[82] Hosseini A., Baeeri M., Rahimifard M., Navaei-Nigjeh M., Mohammadirad A., Pourkhalili N., Hassani S., Kamali M., Abdollahi M. (2013). Antiapoptotic effects of cerium oxide and yttrium oxide nanoparticles in isolated rat pancreatic islets. Hum. Exp. Toxicol..

[83] Cheng Y., Cao X., Liu D. (2026). Copper oxide nanoparticles induce size dependent endothelial damage and cuproptosis via MAPK/NF-κB/NLRP3 signaling pathway. Food Chem. Toxicol..

[84] Liu G., Lv J., Wang Y., Sun K., Gao H., Li Y., Yao Q., Ma L., Kochshugulova G., Jiang Z. (2024). ZnO NPs induce miR-342-5p mediated ferroptosis of spermatocytes through the NF-κB pathway in mice. J. Nanobiotechnol..

[85] Li C., Moro S., Shostak K., O’Reilly F.J., Donzeau M., Graziadei A., McEwen A.G., Desplancq D., Poussin-Courmontagne P., Bachelart T. (2024). Molecular mechanism of IKK catalytic dimer docking to NF-κB substrates. Nat. Commun..

[86] Yazdi A.S., Guarda G., Riteau N., Drexler S.K., Tardivel A., Couillin I., Tschopp J. (2010). Nanoparticles activate the NLR pyrin domain containing 3 (Nlrp3) inflammasome and cause pulmonary inflammation through release of IL-1α and IL-1β. Proc. Natl. Acad. Sci. USA.

[87] Hou S., Li C., Wang Y., Sun J., Guo Y., Ning X., Ma K., Li X., Shao H., Cui G. (2022). Silica Nanoparticles Cause Activation of NLRP3 Inflammasome in-vitro Model-Using Microglia. Int. J. Nanomed..

[88] Adhipandito C.F., Cheung S.-H., Lin Y.-H., Wu S.-H. (2021). Atypical Renal Clearance of Nanoparticles Larger Than the Kidney Filtration Threshold. Int. J. Mol. Sci..

[89] Hauser P.V., Chang H.-M., Yanagawa N., Hamon M. (2021). Nanotechnology, Nanomedicine, and the Kidney. Appl. Sci..

[90] Yu M., Xu J., Zheng J. (2019). Renal Clearable Luminescent Gold Nanoparticles: From the Bench to the Clinic. Angew. Chem..

[91] Li X., Bhullar A.S., Binzel D.W., Guo P. (2022). The dynamic, motile and deformative properties of RNA nanoparticles facilitate the third milestone of drug development. Adv. Drug Deliv. Rev..

[92] Nowak-Jary J., Machnicka B. (2023). In Vivo Biodistribution and Clearance of Magnetic Iron Oxide Nanoparticles for Medical Applications. Int. J. Nanomed..

[93] Hirano M., Maeda K., Matsushima S., Nozaki Y., Kusuhara H., Sugiyama Y. (2005). Involvement of BCRP (ABCG2) in the biliary excretion of pitavastatin. Mol. Pharmacol..

[94] Yang K., Köck K., Brouwer K.L.R., Sugiyama Y., Steffansen B. (2013). Analysis of Hepatic Transport Proteins. Transporters in Drug Development.

[95] Nezasa K.-I., Tian X., Zamek-Gliszczynski M.J., Patel N.J., Raub T.J., Brouwer K.L.R. (2006). Altered Hepatobiliary Disposition of 5 (and 6)-Carboxy-2′,7′-Dichlorofluorescein in Abcg2 (Bcrp1) and Abcc2 (Mrp2) Knockout Mice. Drug Metab. Dispos..

[96] Ouyang B., Poon W., Zhang Y.-N., Lin Z.P., Kingston B.R., Tavares A.J., Zhang Y., Chen J., Valic M.S., Syed A.M. (2020). The dose threshold for nanoparticle tumour delivery. Nat. Mater..

[97] Fröhlich E. (2016). Cellular elimination of nanoparticles. Environ. Toxicol. Pharmacol..

[98] Volatron J., Kolosnjaj-Tabi J., Javed Y., Vuong Q.L., Gossuin Y., Neveu S., Luciani N., Hémadi M., Carn F., Alloyeau D. (2017). Physiological Remediation of Cobalt Ferrite Nanoparticles by Ferritin. Sci. Rep..

[99] Axelsson O., Yousefpour N., Björnberg O., Ekengard E., Lekmeechai S. (2024). Size-dependent renal filtration model explains human pharmacokinetics of a functional nanoparticle: The SPAGOPIX-01 clinical trial. Nanomed. Nanotechnol. Biol. Med..

[100] Iavicoli I., Fontana L., Nordberg G. (2016). The effects of nanoparticles on the renal system. Crit. Rev. Toxicol..

[101] Huang Y., Ning X., Ahrari S., Cai Q., Rajora N., Saxena R., Yu M., Zheng J. (2024). Physiological principles underlying the kidney targeting of renal nanomedicines. Nat. Rev. Nephrol..

[102] Du B., Yu M., Zheng J. (2018). Transport and interactions of nanoparticles in the kidneys. Nat. Rev. Mater..

[103] Poon W., Kingston B.R., Ouyang B., Ngo W., Chan W.C.W. (2020). A framework for designing delivery systems. Nat. Nanotechnol..

[104] Wang Y., Xiao Z., Wang Z., Lee D.Y., Ma Y., Wilhelm S., Wang H., Kim B.Y.S., Jiang W. (2024). Multi-omics approaches to decipher the interactions of nanoparticles and biological systems. Nat. Rev. Bioeng..

